# The Transcriptome Analysis Provides New Insights into Signaling for Bamboo Shoot Development of Sympodial Bamboo

**DOI:** 10.3390/foods14091647

**Published:** 2025-05-07

**Authors:** Shunkai Hu, Mengran Dong, Qirong Guo

**Affiliations:** Co-Innovation Center for Sustainable Forestry in Southern China, Nanjing Forestry University, Nanjing 210037, China

**Keywords:** sympodial bamboo, bamboo shoot development, transcriptome, signaling pathway

## Abstract

Bamboo is a member of the Poaceae family and serves as an important economic resource with various applications, including reforestation, food production, and environmental conservation, due to its rapid growth and renewable nature. Among its various uses, bamboo shoots stand out for their tender texture and delicate flavor, making them a highly sought-after culinary delicacy in many cultures and a key ingredient in global food industries. Despite extensive research on the development of monopodial bamboos, studies focused on the developmental processes of sympodial bamboos, especially regarding their culinary potential, remain limited. This study conducted a comprehensive transcriptomic analysis of sympodial bamboo (*Bambusa* sp.) across six developmental stages (S1–S6) to uncover the molecular regulatory networks governing early bamboo shoot development. The results revealed that 1603 common differentially expressed genes (DEGs) across S1–S6 were enriched in multiple key pathways, with the most significant being plant hormone signaling, MAPK signaling, and Glycolysis/Gluconeogenesis pathways. Co-expression clustering analysis indicated that the Glycolysis/Gluconeogenesis pathway plays a crucial role during the later stages of bamboo shoot development (S5–S6), impacting its texture and flavor—two critical factors determining its culinary quality. Further Weighted Gene Co-expression Network Analysis (WGCNA) highlighted the significant role of the MAPK signaling pathway during early bamboo shoot development and identified key hub genes (*MKK*, *MPK*, *MEKK*) within this pathway, emphasizing their importance in cell division and hormonal coordination. This study provides valuable insights into the molecular mechanisms underlying the rapid growth and exceptional flavor of bamboo shoots and lays the foundation for the genetic improvement of bamboo as a sustainable and nutritious food source, enhancing its value as a premium food ingredient in the global market.

## 1. Introduction

Bamboo, a member of the Poaceae family, is a vital forest and economic resource with diverse applications in reforestation, papermaking, handicrafts, construction, food production, soil and water conservation, and environmental protection [1,2]. As a green and renewable plant, bamboo has garnered global attention for its rapid growth and short rotation cycle, making it a major renewable resource for biomass production [3]. Beyond its ecological and economic significance, bamboo shoots are highly valued for their delicious taste, tender texture, and exceptional nutritional profile. Bamboo shoots are not only a prized delicacy in many cultures but also a versatile ingredient in various cuisines. They offer concentrated nutrients including dietary fiber, plant proteins, vitamins, and key minerals, while containing functional phytochemicals like phenolics and phytosterols. These components contribute to human health by supporting physiological functions and promoting overall well-being, making bamboo shoots an important part of the global food industry [4]. Given the remarkable growth and development characteristics of bamboo, particularly the rapid emergence of bamboo shoots, research into bamboo shoot development has attracted widespread attention [5]. Understanding the mechanisms underlying this rapid growth is crucial not only for bamboo cultivation and utilization but also for enhancing the quality and yield of bamboo shoots, which are a significant food resource.

In recent years, research on the rapid growth and development of bamboo shoots has primarily focused on morphological and anatomical structure, endogenous plant hormone quantification, and high-throughput sequencing. For example, Cui et al. (2012) employed techniques such as proteomics, two-dimensional electrophoresis, and mass spectrometry to reveal that the development, maturation, and aging of Moso bamboo (*Phyllostachys edulis)* culms follow a base-to-top progression [6]. They also identified spatiotemporal specificity in the distribution of endogenous hormones, cell division and elongation, and protein expression levels [6]. Similarly, Herde et al. (2013) conducted expression profiling of bamboo shoots at different heights and regions, uncovering critical roles for pathways such as environmental adaptation, metabolic transformation, and signal transduction, as well as GO terms related to cell growth, division, and differentiation, in the rapid elongation process of bamboo shoots [7]. Research utilized Illumina HiSeq 2000 platform to sequence mixed samples from bamboo shoots at six different heights and mature culms post-leaf expansion. Their analysis identified 10,689 DEGs during rapid growth, with genes related to plant hormones, cell cycle regulation, and cell wall metabolism comprising the majority of DEGs. Among these, 1154 hormone-related genes (11% of DEGs) were identified, with *AUX/IAA* (25) and *LAX/IAA* (10) genes showing significant proportions and predominantly upregulated expression patterns [8,9].

The study of bamboo shoot-to-culm development has long been a focal and challenging area in bamboo research, and shoot apical meristem (SAM) plays a pivotal critical function in accelerated development of bamboo shoots and culms [10]. Research performed transcriptome sequencing of the SAM during the rapid developmental phase of *Moso bamboo*, revealing dynamic expression of auxin signaling and cell cycle-related genes, suggesting that auxin-related genes might regulate the shoot-to-culm transition [11]. Furthermore, alternative splicing events were also found to be involved in this process [11]. Wei et al. (2018) analyzed the transcriptomes of rhizome internodes in *Pseudosasa japonica* and its variant *Pseudosasa japonica var. tsutsumiana* [12]. Compared with the normal species, the slow-growing variant (characterized by shorter, swollen internodes) exhibited significant downregulation of genes associated with cell wall growth, tissue organization, cell division, cell cycle, vesicle transport, and various transport pathways and signaling processes [12]. While an increasing number of studies have explored the molecular mechanisms underlying rapid bamboo shoot development, most focus on monopodial bamboos such as *Moso bamboo* [13]. Sympodial bamboos, such as *Dendrocalamus sinicus*, are characterized by their dense clumping growth and short, thick rhizomes, which differ from the growth patterns seen in monopodial bamboos. Recent advances highlight the unique developmental dynamics of sympodial bamboo shoots [14]. For instance, vascular bundle arrangement in sympodial species exhibits a gradient distribution from the outer to inner culm walls, mirroring the functionally graded structure observed in mature culms, this structural specialization, driven by lignin and cellulose deposition during shoot elongation, directly influences mechanical properties such as tensile strength and flexural rigidity [15]. However, the molecular mechanisms regulating shoot growth phases-from rapid vertical elongation to secondary cell wall thickening-are poorly understood, especially compared to monocarpic monopodial bamboos. This unique growth habit has important implications for bamboo development and cultivation [16]. Further investigation into the shoot development mechanisms of sympodial bamboos is essential for a comprehensive understanding of bamboo growth and its broader applications.

Sympodial bamboos, accounting for over 70% of bamboo species, represent a significant component of the bamboo family. They are predominantly distributed across tropical regions of Southeast Asia, South Asia, Latin America, sub-Saharan Africa, and Pacific island nations [17,18]. Characterized by their tall culms, robust biomass, clumping growth habit, rapid growth, and well-developed rhizome systems. They are also relatively easy to propagate, with flexible nursery methods [19]. Extensive research on sympodial bamboos has focused on fundamental areas such as cultivation techniques, their dual functionality as both timber and edible shoots, and their growth characteristics [20,21,22]. Sympodial bamboos hold versatile applications, primarily in three domains: as a food source, construction material, and ornamental plant. Bamboo shoots from sympodial species are particularly prized as a premium edible product, renowned for their tender texture, crispness, sweet flavor, and rich nutritional content [23]. These shoots are a staple in many culinary traditions and are valued for their high nutritional value, containing 17 essential amino acids, along with vital minerals such as phosphorus, iron, and calcium, as well as vitamins that promote human health [24].

Given the importance of sympodial bamboo shoots in understanding the rapid growth from shoots to culms and their significant edible value, this study aims to elucidate the early developmental mechanisms of these shoots. By performing transcriptome analysis on the shoot tips of sympodial bamboo (*Bambusa* sp.) across six developmental stages (S1–S6), we identified the potential molecular regulatory networks governing the early development of sympodial bamboo shoots. Key signaling pathways associated with rapid shoot growth were uncovered, providing valuable insights for the genetic improvement of bamboos. This work lays a theoretical foundation for enhancing bamboo shoot yield and contributes to the broader understanding of bamboo biology, particularly its role as a sustainable and nutritious food resource.

## 2. Materials and Methods

### 2.1. Plant Materials

The shoot tips of sympodial bamboo (*Bambusa* sp.) were collected from the Bamboo Garden of Nanjing Forestry University. Based on shoot height, the bamboo shoots were categorized into six developmental stages (S1–S6). Shoot tip samples from each developmental stage were immediately snap-frozen in liquid nitrogen and stored at −80 °C for subsequent transcriptome sequencing. Based on length and width, bamboo shoots were divided into six developmental stages. For each stage, three biological replicates were collected, with each biological replicate consisting of 10 samples.

### 2.2. RNA Extraction, Library Construction, and RNA Sequencing

RNA extraction was performed using a plant RNA extraction kit containing DNase according to the manufacturer’s instructions (TIANDZ, Inc., Beijing, China). The quality and concentration of the RNA were assessed using a NanoDrop2000 spectrophotometer (Thermo Fisher Scientific, Waltham, MA, USA), 1.2% agarose gel electrophoresis, and an Agilent 2100 Bioanalyzer (Agilent Technologies, Inc., Santa Clara, CA, USA). After verifying the sample quality, library construction was performed using a series of steps. Eukaryotic mRNA was first enriched with magnetic beads containing Oligo(dT), followed by random fragmentation using Fragmentation Buffer. Using the mRNA as a template, the first cDNA strand was synthesized with random hexamers, and then the second cDNA strand was synthesized by adding buffer, dNTPs, RNase H, and DNA polymerase I, with cDNA purified using AMPure XP beads. The purified double-stranded cDNA underwent end repair, A-tailing, adapter ligation, and fragment size selection using AMPure XP beads, followed by PCR amplification to enrich the cDNA library. After construction, quality control was performed, which included preliminary quantification using Qubit 2.0 and insert size measurement with Agilent 2100, with sequencing only proceeding if the insert size met expectations. The library’s effective concentration was accurately quantified by Q-PCR to ensure it exceeded 2 nM before sequencing. Once the library passed quality control, different libraries were pooled according to the target sequencing data amount, and sequencing was carried out on the BGI DNBSEQ-T7RS platform with a read length of PE150 [25].

### 2.3. Functional Annotation and Enrichment Analysis of Differentially Expressed Genes

High-quality reads were de novo assembled using Trinity (v2.11.0) to produce transcriptome assemblies. The longest transcript for each gene was designated as the unigene. Functional annotation of unigenes was performed using BLASTX against databases including NR (http://ncbi.nlm.nih.gov/, accessed on 12 June 2024), Swiss-Prot (http://www.expasy.ch/sprot, accessed on 21 June 2024), KEGG (http://www.kegg.jp, accessed on 10 July 2024), GO (http://www.geeontology.org, accessed on 10 July 2024), and Pfam (http://pfam.xfam.org/, accessed on 10 July 2024), with an E-value threshold of 1 × 10^5^. Enrichment analyses for GOterms and KEGG pathways were conducted on differentially expressed genes (DEGs), and significant enrichment was visualized using ggplot2 in R (version 4.3.1) [26].

### 2.4. Differential Expression Analysis

Gene expression levels were quantified using RSEM (v1.3.3) based on the mapping results of Bowtie2 (v2.4.4). Differential expression analysis was conducted with DESeq2 or edgeR depending on sample replicates. Thresholds for significant DEGs were set at |log2(fold change)| ≥ 1 and FDR < 0.05. The criteria for differentially expressed genes (|log2(fold change)| ≥1 and FDR < 0.05) balance biological relevance and statistical rigor. |log2(FC)| ≥ 1 (equivalent to ≥2-fold change) filters technical noise and prioritizes genes with potential functional impacts, as historical evidence suggests 2-fold changes often reflect biological significance. FDR < 0.05 controls false positives via multiple testing correction, ensuring < 5% false discovery rate among reported DEGs. These thresholds are widely adopted in transcriptomic studies to ensure reliability and reproducibility. Visualizations, including volcano plots and heatmaps, were generated to illustrate DEG distributions and clustering patterns [27].

### 2.5. WGCNA Analysis

We performed weighted gene co-expression network analysis (WGCNA) to identify biologically relevant gene modules. First, sample clustering (via the hclust function) was applied to exclude outliers. To ensure a scale-free network topology, we determined the optimal soft thresholding power (β) by selecting the smallest value where the scale-free topology model fit index (*R*^2^) exceeded 0.9, as recommended for balancing network connectivity and biological interpretability. The adjacency matrix, transformed using this β value, was converted into a topological overlap matrix (TOM) to minimize spurious correlations. Hierarchical clustering with a TOM-based dissimilarity measure (1-TOM) was then applied, and modules were identified using the dynamic tree-cutting algorithm. To balance specificity and robustness, modules with highly correlated eigengenes (*r* > 0.75, mergeCutHeight = 0.25) were merged, and small modules (<30 genes, minModuleSize = 30) were excluded to reduce noise. Key modules were prioritized based on their correlation coefficients with clinical traits (∣*r*∣ > 0.5, *p* < 0.01). Network visualization was implemented in Cytoscape 3.5.1 [28].

### 2.6. RT-qPCR Analysis

Primers were designed using Vector NTI 10 software, and RT-qPCR was conducted on a Bio-Rad IQ5 real-time PCR system with the SYBR PreMix Ex Taq kit (TaKaRa, Dalian, China) following the manufacturer’s protocol. The thermal cycling program consisted of an initial denaturation at 94 °C for 30 s, followed by 40 cycles of denaturation at 94 °C for 5 s, and annealing/extension for 30 s. *PeGADPH* was selected as internal reference genes. Each experiment included three biological replicates, and relative transcript levels were determined using the 2^−ΔΔCT^ method. Primer sequences are provided in Table S1 [29].

## 3. Result

### 3.1. Transcriptome Data Analysis

**Figure 1:**
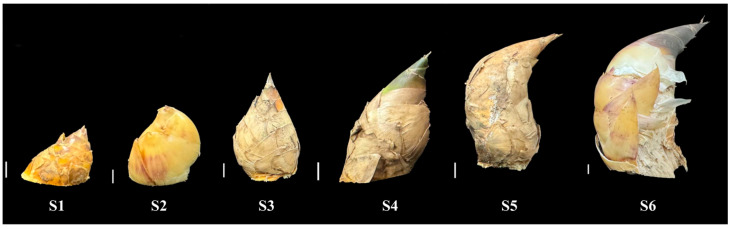
Bamboo shoot phenotypes across developmental stages S1–S6. The white vertical line represents a scale bar, equivalent to 1 cm.

**Figure 2:**
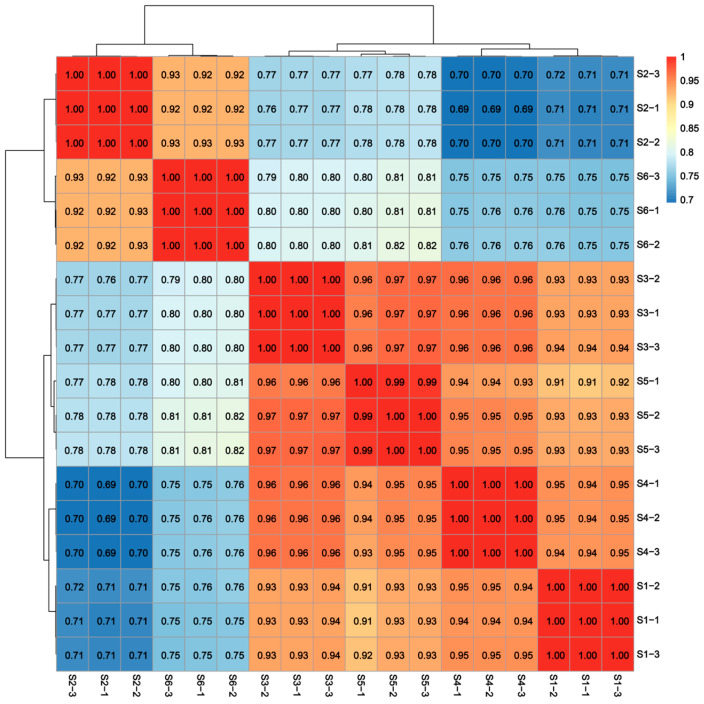
Pearson correlation analysis of S1–S6 development stage of bamboo shoot tips.

The transcriptome sequencing of 18 samples (The six developmental stages (S1–S6, Figure 1) were each replicated three times) was completed, yielding a total of 132.14 Gb of clean data, with each sample providing at least 6.68 Gb of clean data. The Q30 base percentage was consistently above 91.28% (Table S2). Following de novo assembly, a total of 162,760 Unigenes were obtained, with 42,607 Unigenes exceeding 1 kb in length. The clean reads from each sample were aligned to the assembled reference genome, with alignment efficiency ranging from 76.92% to 78.42%. The sample correlation coefficients of the data indicated that both the throughput and quality of the sequencing were sufficiently high to support further analysis (Figure 2).

### 3.2. Function Annotation of Bamboo Shoot Tips

**Figure 3:**
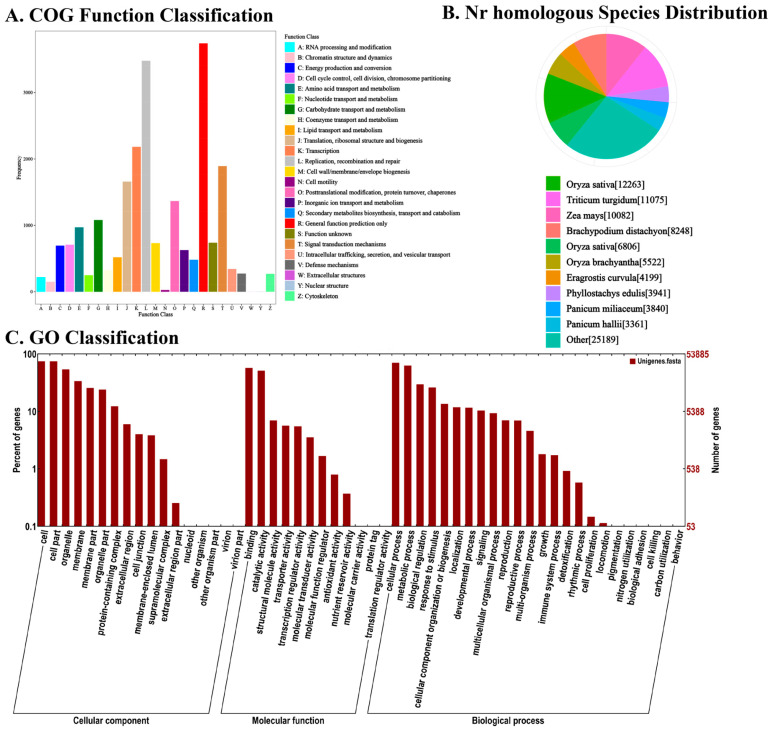
Functional annotation and classification of all unigenes identified in bamboo. (**A**) Determined by Cluster of Orthologous Groups (COG). (**B**) Determined by Non-redundant (NR). (**C**) Determined by gene ontology (GO) databases.

Functional annotation of the assembled genes was conducted using the NR, NT, Swiss-Prot, KEGG, COG, and GO databases (Figure 3). Annotation against the NR database indicated that 12,263 genes shared the highest similarity with *Oryza sativa*, showing the closest phylogenetic relationship among the species examined (Figure 3B). GO term enrichment analysis revealed that, within the Cellular Component category, the genes were predominantly enriched in ‘cell’ and ‘cell part’. In terms of Molecular Function, the genes were most notably associated with ‘binding’ and ‘catalytic activity’. For Biological Process, the majority of genes were enriched in ‘cellular process’ and ‘metabolic process’(Figure 3C). COG classification further highlighted that the annotated genes were largely involved in ‘general function prediction only’ and ‘replication, recombination and repair’(Figure 3A).

### 3.3. GO and KEGG Analysis of Differentially Expressed Genes in Bamboo Shoot Tips at Different Developmental Stages

**Figure 4:**
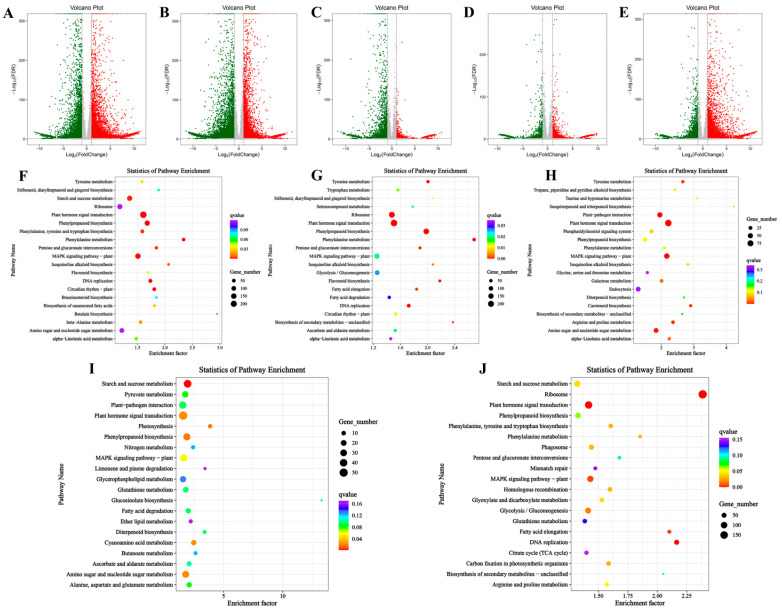
Volcano map and KEGG enrichment analysis of DEGs at different developmental stages. (**A**–**E**): Volcano map of DEGs at different developmental stages. (**A**) KEGG enrichment analysis of DEGs between S1 vs. S2. (**B**) KEGG enrichment analysis of DEGs between S2 vs. S3. (**C**) KEGG enrichment analysis of DEGs between S3 vs. S4. (**D**) KEGG enrichment analysis of DEGs between S4 vs. S5. (**E**) KEGG enrichment analysis of DEGs between S5 vs. S6. F-J: KEGG enrichment analysis of DEGs at different development stages. (**F**) KEGG enrichment analysis of DEGs between S1 vs. S2. (**G**) KEGG enrichment analysis of DEGs between S2 vs. S3. (**H**) S3 vs. S4. (**I**) KEGG enrichment analysis of DEGs between S4 vs. S5. (**J**) KEGG enrichment analysis of DEGs between S5 vs. S6.

In the comparison between the S1 and S2 stages (S1 vs. S2), 9870 genes were upregulated, while 8327 genes were downregulated. In the S2 vs. S3 comparison, 8065 genes were upregulated and 10,637 genes were downregulated. Between the S3 and S4 stages (S3 vs. S4), 2016 genes were upregulated, and 3603 genes were downregulated. In the S4 vs. S5 comparison, 2289 genes were upregulated, and 2146 genes were downregulated. Finally, between S5 and S6 (S5 vs. S6), 6643 genes were upregulated, and 4431 genes were downregulated (Figure 4A–E, Table S3). To investigate the underlying biological mechanisms of bamboo shoot tips development, we performed GO and KEGG enrichment analyses on differentially expressed genes (DEGs) across six developmental stages. GO enrichment analysis revealed significant similarities in the clustering patterns across all stages: in the biological process category, DEGs were predominantly enriched in ‘metabolic process’ and ‘cellular process’; in the cellular component category, DEGs were mainly associated with ‘cell’ and ‘cell part’; and in the molecular function category, the genes were primarily enriched in ‘catalytic activity’ and ‘binding’ (Figure S1). KEGG pathway analysis indicated that DEGs across the various developmental stages were mainly enriched in the MAPK signaling pathway, plant hormone signal transduction, starch and sucrose metabolism, and ribosome-related pathways. Notably, both the MAPK signaling pathway and plant hormone signal transduction were significantly enriched at all stages, highlighting the critical roles of these pathways in bamboo shoot development (Figure 4F–J).

### 3.4. GO and KEGG Enrichment Analysis of Common DEGs at Different Developmental Stages

**Figure 5:**
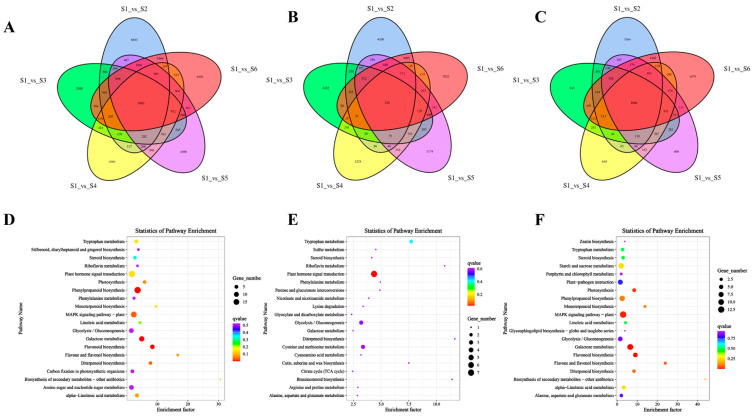
Common DEGs KEGG enrichment analysis at different developmental stages. (**A**–**C**): Venn diagram of common DEGs at different developmental stages (S1 vs. S2, S1 vs. S3, S1 vs. S4, S1 vs. S5, S1 vs. S6). (**A**) Venn diagram of common DEGs. (**B**) Venn diagram of the up-regulated common DEGs. (**C**) Venn diagram of the down-regulated common DEGs. (**D**–**E**): KEGG enrichment analysis of common DEGs at different developmental stages. (**D**) KEGG enrichment analysis of common DEGs. (**E**) KEGG enrichment analysis of up-regulated common DEGs. (**F**) KEGG enrichment analysis of the down-regulated common DEGs.

To further investigate the regulatory mechanisms underlying bamboo shoot development, we conducted GO and KEGG enrichment analyses of commonly differentially expressed genes across different developmental stages. The analysis revealed a total of 1603 commonly differentially expressed genes across the pairwise comparisons of the six stages (S1 vs. S2, S1 vs. S3, S1 vs. S4, S1 vs. S5, S1 vs. S6) (Figure 5A, Table S4). GO enrichment analysis of these common DEGs indicated significant clustering in processes related to ‘cell’, ‘binding’, and ‘cellular process’ (Figure S2A). KEGG pathway analysis highlighted that these genes were primarily enriched in ‘plant hormone signal transduction’, ‘MAPK signaling pathway’, and ‘phenylpropanoid biosynthesis’ (Figure 5D). Further analysis of the upregulated genes within the common DEGs showed enrichment in the same GO categories—‘cell’, ‘binding’, and ‘cellular process’ (Figure 5B and Figure S2B). KEGG enrichment results indicated a prominent role in pathways related to ‘plant hormone signal transduction’, ‘glycolysis/gluconeogenesis’, and ‘cysteine and methionine metabolism’ (Figure 5E). Conversely, downregulated genes were primarily enriched in ‘cell’, ‘binding’, and ‘metabolic process’ according to the GO analysis (Figure 5C and Figure S2C). KEGG analysis of these genes revealed significant enrichment in ‘MAPK signaling pathway’, ‘phenylpropanoid biosynthesis’, and ‘starch and sucrose metabolism’ (Figure 5F).

### 3.5. Co-Expression Clustering Analysis of Genes at Different Development Stages

**Figure 6:**
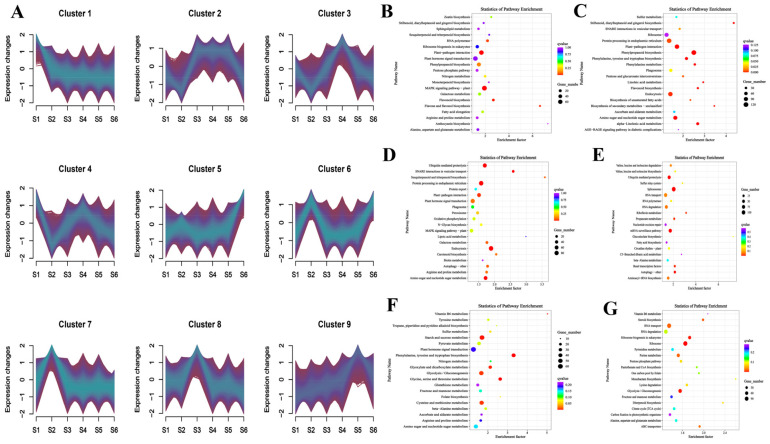
The classification of co-expressed gene clusters at different developmental stages and the main KEGG enrichment pathways at each stage. (**A**) The classification of co-expressed gene clusters. (**B**) Main KEGG enrichment pathways at S1 development stage. (**C**) Main KEGG enrichment pathways at S2 development stage. (**D**) Main KEGG enrichment pathways at S3 development stage. (**E**) Main KEGG enrichment pathways at S4 development stage. (**F**) Main KEGG enrichment pathways at S5 development stage. (**G**) Main KEGG enrichment pathways at S6 development stage.

To explore the gene regulatory mechanisms during different developmental stages of bamboo shoot, we conducted co-expression clustering analysis across six developmental stages. The analysis identified nine distinct expression patterns (Figure 6A, Table S5). For the S1 stage, KEGG pathway enrichment revealed significant clustering in ‘plant hormone signal transduction’, ‘MAPK signaling pathway’, and ‘plant-pathogen interaction’ (Figure 6B). In the S2 stage, enriched pathways were primarily associated with ‘ribosome’, ‘protein processing in the endoplasmic reticulum’, and ‘endocytosis’ (Figure 6C). For the S3 stage, the co-expression analysis highlighted enrichment in ‘ubiquitin-mediated proteolysis’, ‘protein processing in the endoplasmic reticulum’, and ‘plant hormone signal transduction’ (Figure 6D). The S4 stage revealed a strong association with ‘ubiquitin-mediated proteolysis’, ‘spliceosome’, and ‘RNA transport’ (Figure 6E). In the S5 stage, enrichment was observed in ‘starch and sucrose metabolism’, ‘plant hormone signal transduction’, and ‘glycolysis/gluconeogenesis’ (Figure 6F). Finally, the S6 stage was predominantly enriched in pathways related to ‘RNA transport’, ‘RNA degradation’, and ‘ribosome’ (Figure 6G).

### 3.6. Co-Expression Network Analysis Based on WGCNA

**Figure 7:**
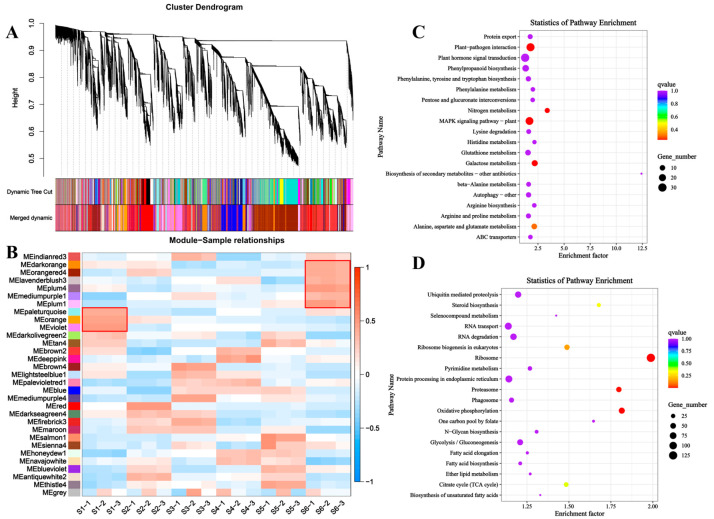
Identification of genes responsive to bamboo shoot tips different development stages by WGCNA analysis. (**A**) Hierarchical cluster tree showing co-expression modules. (**B**) Module–sample association. (**C**) KEGG enrichment analysis of genes involved in the early developmental stages (S1) of bamboo shoot tips. (**D**) KEGG enrichment analysis of genes involved in the final developmental stages (S6) of bamboo shoot tips.

To investigate the regulatory networks involved in bamboo shoot development, we performed a Weighted Gene Co-expression Network Analysis (WGCNA) across six developmental stages of bamboo shoot tips. In WGCNA, modules are defined as clusters of genes with high inter-correlations, with genes within the same module exhibiting strong relationships. A total of 31 distinct modules were identified and color-coded for visualization (Figure 7A). We then conducted a module-sample correlation analysis to identify specific modules that were highly correlated with each developmental stage of bamboo shoot (Figure 7B). The analysis revealed that the modules most closely associated with the early stages of bamboo shoot development were MEpaleturquoise, MEorange, and MEviolet. KEGG pathway enrichment analysis of genes within these modules indicated that early bamboo shoot development is predominantly regulated by signaling pathways such as ‘plant hormone signal transduction’, ‘plant-pathogen interaction’, and ‘MAPK signaling pathway’ (Figure 7C, Table S6). Conversely, the modules most associated with the later stages of bamboo shoot development included MEdarkorange, MEorangered4, MElavenderblush3, MEplum4, MEmediumpurple1, and MEplum1. KEGG enrichment analysis of genes in these modules highlighted significant involvement in pathways related to ‘RNA transport’, ‘RNA degradation’, and ‘protein processing in the endoplasmic reticulum’ (Figure 7D, Table S6).

### 3.7. RT-qPCR Validation of DEGs

**Figure 8:**
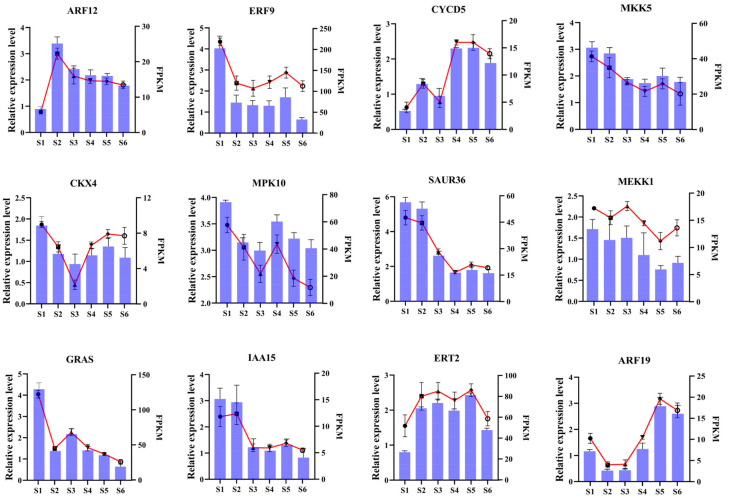
RT-qPCR-based verification of the RNA-seq analysis results. Error bars represent SD, broken lines represent RT-qPCR results, and columns represent RNA-Seq results.

To validate the accuracy of the sequencing data and further explore the expression patterns of genes potentially playing key roles in bamboo shoot development, primers were designed for 12 differentially expressed genes for RT-qPCR verification and expression analysis. The RT-qPCR analysis further validated the RNA-seq findings, as the expression patterns of the differentially expressed genes (DEGs) closely mirrored those observed in the RNA-seq data (Figure 8), thereby confirming the reliability of the transcriptomic results.

## 4. Discussion

In recent years, research on the growth and development of bamboo shoots has primarily focused on aspects such as material growth, anatomical structure, and hormone analysis [30,31,32]. By analyzing the endogenous hormone content and its dynamic changes during different growth stages of Moso bamboo shoots, it has been shown that the rapid growth of bamboo shoots is closely linked to both the levels of endogenous hormones and the ratios between them [33,34]. Anatomical studies reveal that throughout the entire growth process of Moso bamboo shoots, the intercalary meristematic tissue maintains high mitotic activity, while continuous water supply supports rapid shoot elongation, eventually leading to the formation of young bamboo [35]. While the growth phases of Moso bamboo shoots have been extensively studied, little research has been conducted on the development stages of Sympodial bamboo shoots, especially in terms of dividing growth periods based on shoot growth characteristics [36,37]. In this study, transcriptomic analysis was performed on the shoot tips of clumping bamboo shoots at six developmental stages. The results indicated that during clumping bamboo shoot development, differentially expressed genes (DEGs) were primarily enriched in plant hormone signaling and MAPK pathways. In addition, the analysis of DEGs in the later stages of bamboo shoot development showed that differential genes were enriched in the Glycolysis/Gluconeogenesis pathway, which suggested that Glycolysis and Gluconeogenesis play an important role in regulating the later stages of bamboo shoot development.

Consistent with previous findings in monopodial bamboo, our results highlight the central role of hormonal signaling and metabolic reprogramming in sympodial bamboo shoot growth. Transcriptome studies of Moso bamboo (a monopodial species) have likewise identified auxin, ethylene, and energy metabolism pathways as key drivers of rapid shoot development [30]. Our work expands this understanding to a sympodial bamboo, filling a knowledge gap in bamboo biology. The conservation of core pathways between sympodial and monopodial bamboos suggests a fundamental growth program; however, differences in growth habit may lead to nuanced regulatory variations. Sympodial bamboo shoots arise in tight clumps and may sustain their growth phase over a longer period, as indicated by the pronounced upregulation of metabolic genes at later stages in our study. In contrast, Moso bamboo shoots (which emerge from running rhizomes) undergo an intense but relatively brief burst of elongation, drawing heavily on stored resources in a short timeframe [32]. These comparative insights underscore the biological significance of our findings: they reveal a broadly shared developmental strategy across bamboo types, adapted to each species’ growth form and ecological context.

### 4.1. Identification of Hormone Signaling Related DEGs During Bamboo Shoot Tips Development

Plant hormones are crucial regulators of plant growth and development, orchestrating a wide array of physiological processes, from seed germination to fruit ripening. Auxin and ethylene are among the most extensively studied plant hormones, with each playing distinct yet interdependent roles in the regulation of plant growth [38,39,40]. In this study, differential gene expression analysis across various bamboo shoot developmental stages revealed that differentially expressed genes were predominantly enriched in the auxin and ethylene signaling pathways. Auxin, known for its involvement in cell elongation and division, directs the growth of roots and shoots by influencing the formation and patterning of plant tissues. Genes responsive to auxin are divided into three distinct families: AUX/IAA, GH3 (Gretchen Hagen 3), and SAUR (Small Auxin Upregulated RNA) [41]. SAURs are the fastest-responding genes in the auxin signaling pathway, with auxin inducing their expression within minutes, highlighting their critical role in auxin-mediated transcription [42]. Studies have shown that at low auxin concentrations, AUX/IAA proteins bind to auxin response factors (ARFs) and inhibit their transcriptional activity, thus suppressing the auxin response. However, when auxin levels rise, TRANSPORT INHIBITOR RESPONSE1 (TIR1) binds to AUX/IAA proteins, leading to their ubiquitination and the release of ARF transcriptional activity [43]. In this study, to investigate the regulatory mechanisms of auxin signaling during the early developmental stages of bamboo shoots, we focused on genes that were consistently upregulated in comparison to the S1 stage across six developmental periods. The results indicated that differentially expressed genes were predominantly enriched in the ARF gene family (TRINITY_DN16075_c0_g1, TRINITY_DN30381_c0_g1, TRINITY_DN13769_c1_g1, TRINITY_DN3003_c0_g1, TRINITY_DN21008_c0_g1) (Table S4). Furthermore, no members of the ARF family were identified among the genes that were consistently downregulated relative to the S1 stage. This suggests that, during the early stages of bamboo shoot development, the ARF gene family plays a critical role in regulating the auxin signaling pathway. Additionally, differential gene analysis across the six developmental stages revealed upregulation of the auxin efflux carrier family member PIN1 (TRINITY_DN8115_c0_g1) and the SAUR family member (TRINITY_DN91577_c0_g1), indicating the influence of auxin polar transport and early auxin response mechanisms on bamboo shoot development (Table S4).

Ethylene regulates a variety of processes, including fruit ripening, flower senescence, and stress responses, often acting in concert with other hormones to modulate growth under environmental stress. Ethylene signaling in bamboo has a more nuanced role, influencing both growth and maturation processes. While it can promote shoot elongation under certain conditions, it also interacts with auxin to regulate the timing of shoot emergence and maturation [44]. Ethylene’s effect on lignin biosynthesis is particularly important in bamboo, as lignin contributes to the structural integrity of the shoot as it matures. Moreover, ethylene plays a key role in bamboo’s response to environmental stresses such as drought and temperature fluctuations, helping the plant adapt by modulating shoot growth and development in response to unfavorable conditions [45,46]. In this study, by comparing the expression levels of ethylene signaling pathway genes across different bamboo shoot developmental stages, we observed that, during the earliest stages of bamboo shoot development, the expression of genes in the Ethylene-responsive transcription factor (ERF) family was significantly higher (TRINITY_DN83137_c0_g1, TRINITY_DN11466_c0_g1, TRINITY_DN62976_c0_g1, TRINITY_DN10527_c0_g1) (Table S4). Additionally, weighted gene co-expression network analysis (WGCNA) revealed that 18 ERF genes were present in the gene modules associated with the earliest developmental stages of bamboo shoots (Table S6). These findings suggest that ethylene, through the ERF pathway, plays a crucial role in regulating the early development of bamboo shoots.

The concurrent prominence of auxin and ethylene pathways at early stages underscores a coordinated hormonal control during bamboo shoot emergence. The upregulation of multiple ARF genes indicates that auxin signaling is actively driving cell proliferation and elongation in the shoot tip, establishing the rapid growth trajectory characteristic of bamboo. At the same time, the elevated expression of ERF transcription factors suggests that ethylene signaling is engaged to fine-tune this growth. Ethylene likely modulates auxin’s effects—preventing excessive elongation and initiating the first steps of cell maturation (such as subtle cell wall fortification via lignin deposition) even as the shoot extends. This balance between promotion (auxin-driven expansion) and moderation (ethylene-induced adaptation) is crucial for achieving extraordinary growth rates without compromising structural integrity. Early ethylene signaling may also prime the developing shoot for environmental challenges, ensuring resilience as it emerges from the soil. Notably, such hormone crosstalk aligns with observations in Moso bamboo, where dynamic changes in auxin and other hormones correlate with phases of rapid shoot elongation [33,34]. By orchestrating growth (auxin) and stress-readiness (ethylene) simultaneously, the bamboo shoot optimizes its development for both speed and survival.

### 4.2. MAPK Signaling Pathway Regulates Bamboo Shoot Tips Development

In plants, the MAPK signaling pathway plays a crucial role in regulating growth and development, programmed cell death, and responses to various abiotic stresses (such as cold, heat, reactive oxygen species, UV radiation, and drought) as well as biotic stresses (including pathogen and insect attacks) [47,48]. MAPK pathways are involved in multiple developmental processes, including stomatal development, organ abscission, meristem maintenance, inflorescence architecture, and root development. In *Arabidopsis*, the receptors ERECTA (ER), ERECTA-like 1 (ERL1), and ERL2 are essential for promoting localized cell proliferation, thereby determining inflorescence structure, organ shape, and size. The YDA MKK4/MKK5-MPK3/MPK6 cascade, which operates downstream of the ER receptors, regulates local cell proliferation, thus shaping plant organ morphology [49,50]. Loss of function in MPK3/MPK6 or MKK4/MKK5 leads to shortened pedicels and clustered inflorescences. These findings suggest that the YDA–MKK4/MKK5–MPK3/MPK6 MAPK cascade plays a critical role in regulating local cell division downstream of the ER receptors. Furthermore, the YDA-MKK4/MKK5-MPK3/MPK6 cascade is a key signaling pathway involved in asymmetric cell division during embryonic development [51]. While extensive research has been conducted on MAPK pathways in model plants like *Arabidopsis*, studies specifically focusing on bamboo are limited. However, transcriptome-wide analyses have identified that hormone signaling and MAPK signaling pathways are among the most enriched pathways in transcription factor-regulated networks during bamboo culm development [52]. This suggests that MAPK pathways may play a significant role in regulating the growth and development of bamboo, particularly in processes such as cell wall formation and response to environmental stresses.

In this study, differential gene expression analysis across various developmental stages revealed a significant upregulation of genes associated with the MAPK signaling pathway during the S1 stage compared to other stages. Co-expression cluster analysis further demonstrated that genes involved in the MAPK signaling pathway were notably enriched in the S1 stage. To further investigate the potential role of the MAPK pathway during this stage, we conducted WGCNA analysis and identified key MAPK pathway genes within the S1 gene module. The analysis revealed nine hub genes, including MKK (TRINITY_DN6930_c0_g1, TRINITY_DN17272_c0_g1, TRINITY_DN7778_c0_g1), MPK (TRINITY_DN23216_c0_g1, TRINITY_DN9469_c0_g2, TRINITY_DN1082_c1_g2, TRINITY_DN17105_c0_g1), MEKK (TRINITY_DN26155_c0_g1), and MAPKKK (TRINITY_DN42786_c1_g1) (Table S6). These findings suggest that the MAPK signaling pathway plays a crucial role in regulating cell division at the shoot tip and coordinating hormonal regulation during the early stages of sympodial bamboo shoot development.

The prominent activation of MAPK signaling in the earliest developmental stage highlights its importance as an integration point for growth and environmental cues. The enrichment of MAPK-related genes in stage S1 implies that as soon as the bamboo shoot initiates development, it engages MAPK cascades to drive the proliferation of meristematic cells. Given that MAPK pathways can mediate crosstalk between hormone signals and stress responses, their early activation may ensure that signals from auxin and ethylene are effectively translated into the cellular actions needed for rapid growth. In the dark, subterranean conditions where bamboo shoots initially grow, MAPK signaling might also respond to mechanical stress (e.g., pushing through soil) or hypoxic conditions, thereby safeguarding the meristem’s functionality. The identification of multiple MAPK components as hub genes in our co-expression network underscores the pathway’s regulatory centrality. This finding is in line with recent studies on other bamboo species; for instance, a transcriptome analysis of *Dendrocalamus sinicus* (a giant sympodial bamboo) found that MAPK-related regulators were among the core drivers of culm development [52]. Our results extend this concept to the early shoot stage, suggesting that a conserved MAPK-centered module orchestrates the initial growth spurt of bamboo shoots. Thus, the MAPK pathway in bamboo not only echoes its known roles in model plants but also appears to be tailored to support the unique demands of bamboo’s fast and tall growth habit.

### 4.3. Glycolysis/Gluconeogenesis Pathway Is Involved in Bamboo Shoot Development

The Glycolysis/Gluconeogenesis pathways are essential for plant growth and development by regulating energy metabolism and carbon homeostasis. Glycolysis, which converts glucose into pyruvate, provides ATP and NADH, crucial for energy production during periods of rapid growth or low oxygen availability. This pathway supports processes such as cell division, biosynthesis, and stress responses, and generates precursors for amino acids, lipids, and secondary metabolites, all vital for plant development [53,54]. Conversely, gluconeogenesis, which synthesizes glucose from non-carbohydrate precursors like pyruvate, becomes active during periods of low photosynthesis, such as at night or under stress conditions. It ensures a continuous supply of glucose, supporting cellular functions and carbon storage, especially during times when external carbon fixation is minimal [55,56]. The interplay between these two pathways is tightly regulated to balance energy production and carbon storage efficiently, ensuring that plants can adapt to fluctuating environmental conditions. This coordination is critical for optimal growth, especially in maintaining energy supply during dark periods or stress conditions, and for sustaining metabolic processes when photosynthesis is not actively generating carbon [57]. In this study, we analyzed differentially expressed genes consistently upregulated across the S1–S6 developmental stages of bamboo shoots. The results indicated that these genes were significantly enriched in the Glycolysis/Gluconeogenesis pathway. Co-expression cluster analysis further revealed that, during the S5 and S6 developmental stages, genes in the Glycolysis/Gluconeogenesis pathway were notably upregulated. To explore this further, we conducted WGCNA analysis and identified key hub genes within the S6 gene module. The findings highlighted that these hub genes were enriched in the Glycolysis/Gluconeogenesis pathway, suggesting a critical regulatory role of this pathway in bamboo shoot growth and development, particularly during the later stages (Table S6). Furthermore, the screening of hub genes within the Glycolysis/Gluconeogenesis pathway identified 56 genes in the S6 module. Co-expression analysis at the S6 stage revealed that the differentially expressed genes TRINITY_DN62359_c0_g1, TRINITY_DN1007_c0_g1, TRINITY_DN16431_c0_g1, TRINITY_DN1881_c0_g1, and TRINITY_DN1949_c2_g1 were all upregulated, with TRINITY_DN62359_c0_g1 showing upregulation across all six developmental stages (S1–S6) (Table S6). These results suggest that TRINITY_DN62359_c0_g1 may play a key regulatory role in the developmental processes of bamboo shoots.

The stage-specific enhancement of glycolytic and gluconeogenic activity points to a developmental transition from a signaling-centric program to a metabolism-centric program as the bamboo shoot matures. In earlier stages, hormonal signals and MAPK cascades dominate to kick-start growth, but as the shoot reaches stages S5–S6, the demand for energy and biosynthetic building blocks surges. The marked upregulation of glycolysis-related genes in these later stages indicates that the shoot tip is generating large amounts of ATP and metabolic intermediates to support rapid cell expansion, cell wall synthesis, and other growth processes. At the same time, the activation of gluconeogenesis suggests the utilization of stored carbon reserves to maintain a steady glucose supply when photosynthetic output is still limited. Bamboo shoots, especially in sympodial species, may prolong their heterotrophic growth until a substantial height is reached. Our findings align with the known strategy of bamboo, wherein a burst of elongation is fueled by previously accumulated resources [32]. By the time the shoot emerges above ground and its young leaves become photosynthetically active, the foundation—built on internal energy reserves—has already been laid through these metabolic pathways. These metabolic shifts also carry important implications for the culinary quality and harvest timing of edible bamboo shoots. Early developmental stages, characterized by vigorous cell division and expansion, generally yield shoots with tender texture and higher water content, which are ideal for consumption. As the shoot progresses to later stages (S5–S6) and metabolic and structural genes ramp up, there is likely an increase in fiber and a change in the shoot’s biochemical composition. The onset of extensive gluconeogenesis and glycolysis might coincide with the accumulation of sugars and other flavor-related compounds, potentially enhancing the sweet taste up to a point, but continued development can lead to the conversion of sugars into structural carbohydrates, reducing palatability. Ethylene’s role in promoting lignification [44] further suggests that if a shoot is left to develop too long, it will become firmer and less edible. Understanding the timing of these molecular changes provides practical insight: to maximize tenderness and flavor, bamboo shoots should be harvested before the late developmental transition when energy metabolism peaks and significant lignification begins. From an agricultural perspective, managing soil nutrients and water to support the plant’s energy needs during early rapid growth could yield more robust shoots, but producers must balance this with timely harvest to ensure quality. In summary, the integration of signaling and metabolic pathways revealed by our study not only elucidates the developmental biology of sympodial bamboo shoots but also guides practices to optimize their growth and utilization as a nutritious food resource.

### 4.4. Association Between Key Signal Pathways in Early Development of Bamboo Shoots and Edible Traits

The early developmental stages of bamboo shoots are essential for their edible quality, as rapid cell division, nutrient accumulation, and secondary metabolite synthesis during this phase directly affect texture, flavor, and nutritional content. WGCNA analysis identified four key pathways—nitrogen metabolism, galactose metabolism, phenylpropanoid biosynthesis, and aromatic amino acid biosynthesis—that regulate these processes. Nitrogen metabolism is pivotal for synthesizing amino acids, proteins, and nucleotides, which are essential for bamboo shoot growth. High nitrogen assimilation activity in early development likely promotes the accumulation of free amino acids, such as glutamate and asparagine, which contribute to the characteristic umami taste of bamboo shoots [58]. Glutamate, a precursor of γ-aminobutyric acid (GABA), may also enhance the nutritional profile by providing potential neuroprotective benefits [59].

Galactose metabolism plays a crucial role in the texture and postharvest quality of bamboo shoots [60]. This metabolic process primarily involves the conversion of galactose to UDP-galactose, an essential substrate for the synthesis of cell wall polysaccharides such as pectins and hemicelluloses [61]. These polysaccharides contribute to the structural integrity and flexibility of the cell wall, directly influencing the texture of bamboo shoots [62]. Pectins, in particular, are well known for their ability to maintain cell wall flexibility, which is critical for preserving the tender and crisp texture required in bamboo shoots [63]. During the early stages of bamboo shoot development, sustained galactose metabolism is vital for maintaining cell wall integrity and delaying the lignification process, where the cell wall becomes rigid and woody. This is especially important for bamboo shoots, as excessive lignification can lead to a fibrous and tough texture, reducing their palatability [5]. Studies in other plants, such as apples, have shown that galactose metabolism helps maintain fruit crispness by stabilizing pectin content in the cell wall, thus delaying the softening process during storage [64]. Similarly, in bamboo shoots, continuous galactose metabolism may contribute to a firmer yet tender texture, crucial for maintaining quality during postharvest storage [65]. On the other hand, disruptions in galactose metabolism can lead to abnormal cell wall thickening, as seen in *Arabidopsis* mutants with impaired galactose mutarotase [66]. These disruptions result in texture changes, making bamboo shoots less tender and more fibrous, negatively affecting their edible quality. Therefore, the regulation of galactose metabolism is closely linked to the texture and overall quality of bamboo shoots. By maintaining proper galactose metabolism during bamboo shoot growth and development, it is possible to optimize their texture, ensuring they remain tender, crisp, and flavorful.

Phenylalanine, tyrosine, and tryptophan serve as hubs linking primary and secondary metabolism [67]. Tryptophan-derived auxin regulates cell elongation, critical for rapid shoot growth and tenderness. In bamboo shoots, auxin peaks during early elongation, driving rapid cell expansion [68]. Meanwhile, phenylalanine fuels phenylpropanoid biosynthesis, while tyrosine is a precursor for melanin, a pigment implicated in postharvest browning [69]. Excessive tyrosine decarboxylase activity can exacerbate browning through dopamine oxidation, while controlled accumulation of tyrosine-derived volatiles (e.g., tyrosol) may enhance flavor complexity [70]. These findings highlight the necessity of maintaining a balance between the growth-promoting, flavor-forming, and stress-responsive roles of aromatic amino acid

Together, these metabolic networks integrate nitrogen, carbohydrate, and phenolic metabolism to regulate bamboo shoot growth, flavor, and postharvest quality. Understanding this interplay offers insights for improving bamboo shoot cultivation practices to optimize texture, flavor, and nutritional value.

## 5. Conclusions

This study provides valuable insights into the molecular mechanisms regulating the early development of sympodial bamboo shoots. Our findings reveal that plant hormone signaling and MAPK pathways drive rapid growth in the early stages (S1–S4), while glycolysis and gluconeogenesis pathways become more prominent in later stages (S5–S6) to support energy demands and shoot maturation. These metabolic shifts have significant implications for bamboo shoot quality, as early growth stages are associated with tender, flavorful shoots, ideal for culinary use. However, as shoots mature, the activation of phenylpropanoid biosynthesis leads to lignin accumulation, resulting in tougher, more fibrous shoots with reduced sweetness.

The practical implications of these findings emphasize the importance of optimizing harvest timing to preserve bamboo shoot texture, flavor, and nutritional value. By harvesting bamboo shoots before the late developmental stages, when lignification begins, it is possible to retain their desired culinary qualities. Understanding key metabolic processes like glycolysis and galactose metabolism provides opportunities to improve postharvest quality and nutrition. Overall, this study highlights how integrating molecular insights can inform cultivation practices to enhance the quality of bamboo shoots as a nutritious and flavorful food source.

## Data Availability

The transcriptome data has been deposited in the NCBI SRA database with the accession number PRJNA1252968.

## Supplementary Materials

The following supporting information can be downloaded at: https://www.mdpi.com/article/10.3390/foods14091647/s1, Figure S1. GO enrichment analysis of DEGs at different development stages; Figure S2. Common DEGs GO enrichment analysis at different developmental stage; Table S1. Primer sequences; Table S2. Quality assessment of clean data; Table S3. DEGs between different development stages; Table S4. Common DEGs at different developmental stages; Table S5. Co-expression clustering analysis of genes at different development stages; Table S6. Identification of genes responsive to bamboo shoot tips different development stages by WGCNA analysis.
