# Supplementary material for: The Transcriptome Analysis Provides New Insights into Signaling for Bamboo Shoot Development of Sympodial Bamboo
Source: Foods. 2025 May 7;14(9):1647. doi: 10.3390/foods14091647 (PMC12072057; doi:10.3390/foods14091647)
Supplement: Supplementary file 1 [file foods-14-01647-s001.zip › supplemental Figure.pdf]

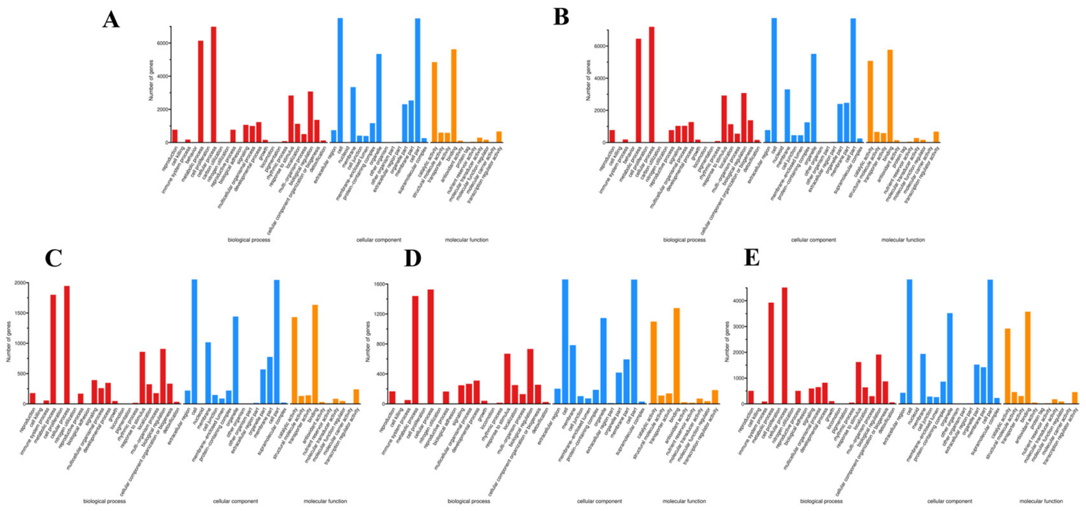

Figure S1. GO enrichment anaylsis of DEGs at different development stages. A-E: GO enrichment anaylsis of DEGs at different development stages. (F) S1 vs. S2. (G) S2 vs. S3. (H) S3 vs. S4. (I) S4 vs. S5. (J) S5 vs. S6.

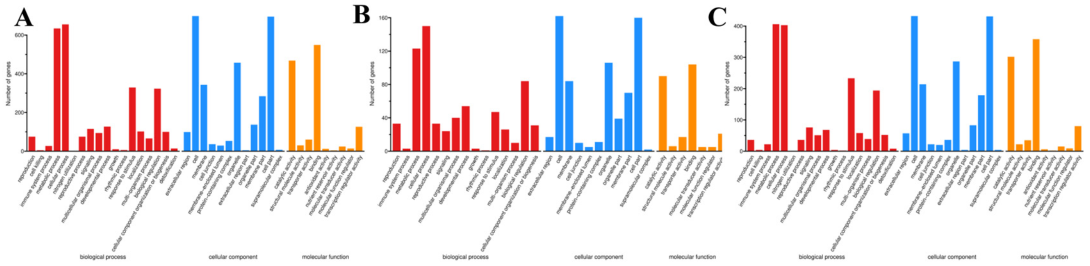

Figure S2. Common DEGs GO enrichment analysis at different developmental stage. A-C: GO enrichment analysis of common DEGs at different developmental stages. (A) GO enrichment analysis of common DEGs. (B) GO enrichment analysis of up-regulated common DEGs. (C) GO enrichment analysis of the down-regulated common DEGs.
